# Recent Progress in Photocatalysis Mediated by Colloidal II-VI Nanocrystals

**DOI:** 10.1002/ijch.201200073

**Published:** 2012-12-13

**Authors:** Molly B Wilker, Kyle J Schnitzenbaumer, Gordana Dukovic

**Affiliations:** [a] Department of Chemistry and Biochemistry, University of Colorado BoulderBoulder, CO 80309, USA

**Keywords:** charge transfer, hydrogen generation, photocatalysis, quantum dots, semiconductor nanocrystals

## Abstract

The use of photoexcited electrons and holes in semiconductor nanocrystals as reduction and oxidation reagents is an intriguing way of harvesting photon energy to drive chemical reactions. This review focuses on recent research efforts to understand and control the photocatalytic processes mediated by colloidal II-VI nanocrystalline materials, such as cadmium and zinc chalcogenides. First, we highlight how nanocrystal properties govern the rates and efficiencies of charge-transfer processes relevant to photocatalysis. We then describe the use of nanocrystal catalyst heterostructures for fuel-forming reactions, most commonly H_2_ generation. Finally, we review the use of nanocrystal photocatalysis as a synthetic tool for metal–semiconductor nano-heterostructures.

## 1. Introduction

**Figure 1:**
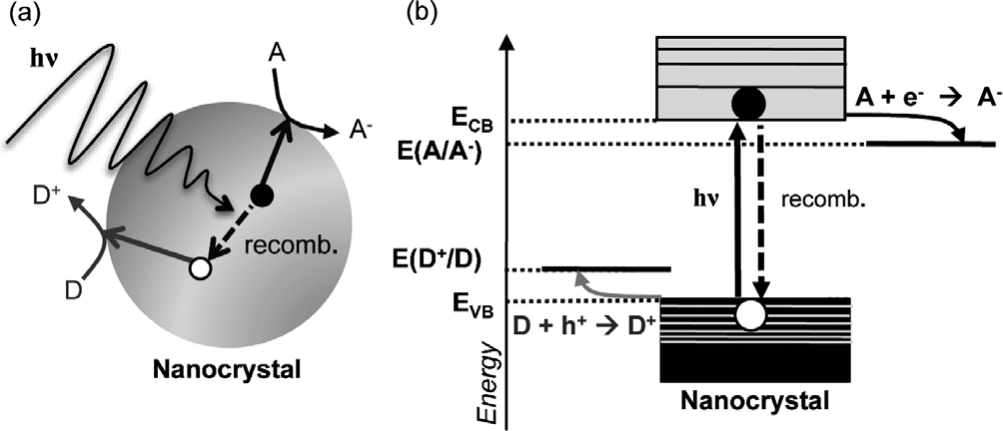
(a) Schematic representation of a photocatalytic reaction mediated by a semiconductor nanocrystal. Photoexcited electrons migrate to the surface and reduce an electron acceptor (A), while the holes oxidize an electron donor (D). This process is in competition with electron–hole recombination pathways. (b) Energy level diagram for a photocatalytic reaction, indicating energetic requirements for valence and conduction band edges (*E*_VB_ and *E*_CB_) with respect to reduction potentials of the donor and acceptor (*E*(D^+^/D) and *E*(A/A^−^)).

Colloidal semiconductor nanocrystals are well known for their remarkably tunable optical properties, which depend on structural parameters, most notably nanocrystal size.^1^–^3^ Nanocrystal photophysics – the behavior and dynamics of their photoexcited states – has been a subject of intense research for three decades.^4^–^14^ This work has uncovered many notable phenomena, such as robust photoluminescence useful for biological labeling^15^–^17^ and multiple exciton generation that could increase efficiencies of solar cells.^12^,^18^–^20^ Recent years have witnessed a growth in research exploring nanocrystal photochemistry, particularly in the case of II-VI chalcogenides, such as CdX and ZnX where X=S, Se, and Te.^21^–^43^ Processes of interest for this review use photoexcited electrons and holes to reduce and oxidize, respectively, species on the nanocrystal surface or in solution (Figure 1a). Ideally, photochemistry occurs without chemical changes to the nanocrystal. For this reason, the term photocatalytic^44^ is often employed to describe such reactions.^45^–^47^ The photocatalytic reactions we discuss herein are characterized by favorable thermodynamics (i.e., sufficient driving force for reduction and oxidation) and kinetic competitiveness with electron–hole recombination pathways (Figure 1b).

Several characteristics of II-VI semiconductor colloidal nanocrystals make them particularly interesting photocatalysts: (i) They absorb in the visible region, so they are able to harvest a significantly higher portion of the solar spectrum than the UV-absorbing oxides. (ii) Chalcogenide nanocrystals are strong light absorbers, with molar absorptivities of about 10^5^–10^7^ m^−1^ cm^−1^.^38^,^48^–^50^ (iii) Their band edges, redox potentials, and absorption spectra are readily tunable. These parameters are determined by nanocrystal composition, size, and shape. (iv) At the size scale of several nanometers, photoexcited carriers have facile access to the surfaces where they can be utilized. (v) Nanocrystal surfaces can be capped with a rich variety of ligands, enabling either aqueous or organic solubility and providing selectivity of functional groups for interaction with molecular species in solution.^51^ These qualities are being employed in nanocrystal-based photocatalytic systems that generate fuels,^23^,^26^,^29^,^33^ as well as for the light-driven synthesis of nano-heterostructures.^21^–^32^

Photochemical redox reactions of nanoscale semiconductors have been studied since the early 1980s.^52^ Examples from that time period include photoreduction of viologens mediated by CdS nanoparticles,^53^–^57^ generation of H_2_ and/or O_2_ from H_2_O using CdS colloidal systems coupled to small metallic particles,^53^,^58^–^60^ and a demonstration that quantum confinement in small semiconductors can enhance photoredox chemistry.^61^ It was quickly recognized that particle degradation by photo-oxidation presented a major challenge for the field of nanoscale semiconductor photocatalysis.^55^,^62^–^64^ This problem was previously well known in bulk chalcogenides.^65^–^67^ Improvements in the understanding of excited-state properties and advances in synthetic methods achieved throughout the last three decades^4^–^14^,^51^,^68^–^73^ provide new opportunities for the exploration of nanocrystal photocatalysis.

This review focuses on the developments in photocatalysis mediated by colloidal II-VI chalcogenide nanocrystals (composed of CdX and ZnX, where X=S, Se, and Te) that have occurred in the last five years. We specifically consider solution-synthesized, solution-phase II-VI chalcogenide nanocrystalline materials in the quantum confinement size regime. We omit discussion of photocatalysis using nanoscale but not colloidal chalcogenides, as well as the use of oxide photocatalysts. These topics have been reviewed elsewhere.^45^,^74^–^79^ First, we briefly overview insights into nanocrystal photocatalysis obtained from measurements of charge-transfer dynamics between nanocrystals and well-known electron donors or acceptors. Next, we focus on the use of nanocrystals for photocatalytic fuel formation under visible irradiation. This process is enabled by coupling inorganic or biological co-catalysts to light-harvesting nanocrystals. Finally, we describe how photocatalytic metal deposition can be used as a synthetic tool.

## 2. Insights from Nanocrystal Charge-Transfer Dynamics

Photocatalytic reactions that are of potential practical importance, such as fuel generation, are complex multielectron processes. Their overall kinetics depend not only on the nanocrystal properties but also on the kinetics of catalysis. To isolate the role of nanocrystal properties in processes relevant to photocatalysis, it is useful to study the dynamics of relatively simple one-electron redox reactions that have products with spectroscopic signatures in the visible wavelength range. Examples include measurements of rates of electron transfer (ET) to acceptors such as rhodamine B,^80^–^83^ anthraquinone (AQ),^84^–^87^ methylene blue (MB^+^),^88^–^90^ and viologens–most commonly methyl viologen (MV^2+^).^91^–^101^ Acceptors with spectroscopic signatures for the oxidized species, used to measure rates of hole transfer (HT), include phenothiazine^102^,^103^ and *p*-phenylenediamine.^104^ In addition, important insights have been obtained from measurements of ET dynamics from nanocrystals to metal oxides.^105^–^111^

The charge-transfer rates discussed in this section are most commonly measured by ultrafast transient absorption (TA)^112^,^113^ and time-resolved photoluminescence (PL).^114^ The charge-transfer reactions compete with internal electron–hole recombination pathways and shorten carrier lifetimes. For II-VI nanocrystals, signals associated with photoexcited electrons are considerably easier to detect than those that correspond to the holes. The band-gap bleach in the visible region, which is a very prominent spectroscopic feature in nanocrystal TA, is primarily due to electron population in the 1S conduction-band state.^8^,^12^ To deduce hole dynamics, one can compare TA and PL decay signals because the PL signal strength reflects both the electron and hole populations.^115^,^116^ Hole signals can also be detected directly by intraband transitions in the IR region.^117^–^119^ In combination with such experiments, the spectroscopic signature of an oxidized dye can be used to monitor the hole dynamics upon transfer out of the nanocrystal.^102^–^104^ In this section, we describe the theoretical framework for understanding charge-transfer dynamics in nanocrystals, discuss examples of how such processes depend on electronic coupling and driving force, and illustrate how band engineering can be used to control charge-transfer efficiencies. We end with a comparison of reported charge-transfer rates and the rates of other carrier decay processes in nanocrystals, demonstrating the feasibility of nanocrystal photocatalysis.

### 2.1. Theoretical Framework for Charge Transfer from Photoexcited Nanocrystals

To understand the factors that control the rates of charge transfer from photoexcited nanocrystals, Marcus theory of ET^120^,^121^ is commonly invoked.^10^,^83^,^85^,^86^,^97^,^99^,^108^,^122^–^124^ Within this framework, the rate constant for ET (*k*_ET_) from an electron donor (D) to an electron acceptor (A) is given by Equation (1):
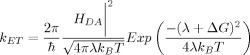
(1)

The key parameters in this expression are *H*_DA_, the electronic coupling between D and A; *λ*, the reorganization energy (energy cost of having the D–A pair in the nuclear geometry of the charge-separated state); and Δ*G*, the driving force (the free energy difference between the photoexcited D–A pair and the charge-separated state). Equation (1) is suitable to describe ET from the lowest lying 1S conduction-band state of the nanocrystal to LUMO levels of molecular acceptors (Figure 1b). For ET into metal oxide conduction bands, the density of acceptor states is accounted for in a more complex formulation of Equation (1).^108^,^122^ Similar modifications may be needed for HT from more densely spaced nanocrystal valence-band states. The nanocrystal contribution to *λ* is thought to be small because the nuclear positions are not strongly perturbed by ET, while the acceptor contribution can vary widely, depending on the choice of the acceptor and the solvent.^83^,^85^,^86^,^97^,^108^ The ability to control both the surface chemistry and the band-edge potentials of nanocrystals allows for tuning of *H*_DA_ and Δ*G*, and therefore, control of charge-transfer rates.

### 2.2. Effect of Electronic Coupling on Measured ET Rates

The electronic coupling, *H*_DA_, between a nanocrystal donor and a charge acceptor can be tuned by controlling their binding interaction. The interaction can range from direct adsorption onto the crystal surface to attachment through a molecular linker, which can act as a tunneling barrier. Electron injection rates from CdSe quantum dots (QDs) to TiO_2_ were found to be three times faster when the two were attached by direct adsorption rather than through a molecular linker.^109^ In a system consisting of CdS QDs linked to TiO_2_ by mercaptocarboxylate (S^−^–(CH_2_)_*n*_–COO^−^) bridges, faster charge transfer was observed with fewer CH_2_ units in the chain.^110^ Similarly, in thin films made from CdSe QDs bound to poly(viologen) by S^−^–(CH_2_)_*n*_–COO^−^ linkers, ET rates decreased with increasing chain length.^100^ The extent of electron delocalization in the linker molecule can also affect ET rates. For example, the rates of ET from CdS QDs to TiO_2_ through 3-mercaptopropionic and 4-mercaptobenzoic acids were comparable, indicating that the increased electronic coupling through the phenyl group compensated for the larger spacing between the donor and acceptor.^111^ Similar dependence of electronic coupling on both the distance and electronic properties of the linker material has been seen for electron injection from molecular adsorbates to TiO_2_ nanoparticles.^122^ These examples suggest that nanocrystal–acceptor interfaces can be designed to facilitate strong electronic coupling and optimize ET rates.

### 2.3. Effect of Δ*G* on Measured ET Rates

**Figure 2:**
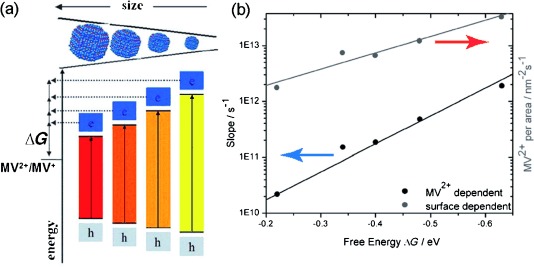
a) Schematic representation of how changes in CdSe QD sizes affect Δ*G* for ET in the CdSe–MV^2+^ system. With the decrease in nanocrystal size, the electron and hole become more confined, widening the band gap and shifting the band-edge potentials. (b) The dependence of measured ET rates on Δ*G*. The rates on the left axis (data marked with the blue arrow) have been adjusted to account for the surface area of differently sized CdSe QDs. Rates on the right axis (red arrow) have been adjusted for the average number of MV^2+^ molecules per CdSe area. Both data sets follow the exponential dependence on Δ*G* predicted by Eq. (1). Adapted with permission from Ref. [99]. Copyright 2011, Wiley.

**Figure 3:**
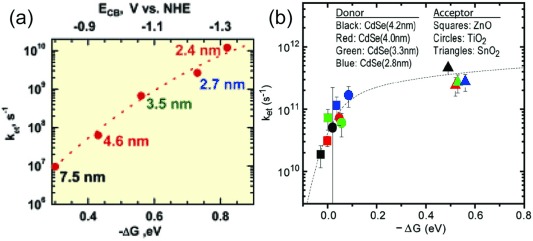
(a) Measured ET rates from CdSe QDs of various sizes to molecularly linked TiO_2_ as a function of Δ*G* illustrate the exponential dependence predicted by Eq. (1). Reproduced with permission from Ref. [106]. Copyright 2007, ACS. (b) Measured ET rates from CdSe QDs of various sizes to three metal oxides as a function of Δ*G*. Due to increased electronic coupling and the density of metal oxide acceptor states, the rate of ET demonstrates an exponential dependence on Δ*G* for lower values of Δ*G*, but transitions to a non-exponential dependence at higher values of Δ*G*. Adapted with permission from Ref. [108]. Copyright 2011, PNAS.

While the reduction potential of a particular electron acceptor is often fixed, changing the size of the nanocrystal alters its band-edge potentials.^2^,^4^,^125^ When not directly measured,^126^ nanocrystal redox potentials are most commonly estimated by applying quantum confinement corrections to the bulk band-edge potentials of the materials in question.^3^,^82^,^99^,^102^,^106^,^108^,^123^ In the materials discussed herein, most of the band-gap shift is allocated to the conduction-band levels because electrons are considerably lighter than holes. The shift of band-edge potentials with nanocrystal size and the corresponding change in Δ*G* for the example of ET from CdSe to MV^2+^ are depicted in Figure 2a. The resulting effects on charge-transfer rates have been demonstrated for ET from CdSe QDs to molecular species such as an adsorbed Re–bipyridyl complex,^127^ adsorbed MV^2+^,^99^ and covalently linked thiol-functionalized fullerene.^123^ The rate of ET to MV^2+^ has an exponential dependence on Δ*G* as predicted by Equation (1) (Figure 2b).^99^ Likewise, the electron injection rate from CdSe QDs to molecularly linked TiO_2_ increased exponentially with decreasing QD size (Figure 3a).^106^ The direct adsorption of CdSe QDs on TiO_2_, SnO_2_, and ZnO led to more complex behavior due to stronger electronic coupling (Figure 3b).^108^ As expected from the observations described in Section 2.2., ET rates range between 10^10^ and 10^12^ s^−1^ for directly adsorbed acceptors (Figure 3b) compared with 10^7^–10^10^ s^−1^ for molecularly linked acceptors (Figure 3a). Figure 3b illustrates the role of the density of acceptor states in the highly coupled regime. For smaller values of Δ*G*, the rate of ET behaved according to the traditional exponential form of a molecular Marcus picture. As Δ*G* increased, however, the rate of ET transitioned to being dominated by the density of acceptor states, resulting in a deviation from the behavior predicted by Equation (1).^108^ For this reason, the Marcus inverted region was not observed and an additional driving force over 200 meV did not significantly improve or diminish ET rates.

### 2.4. Use of Wavefunction Engineering to Control ET Efficiency

**Figure 4:**
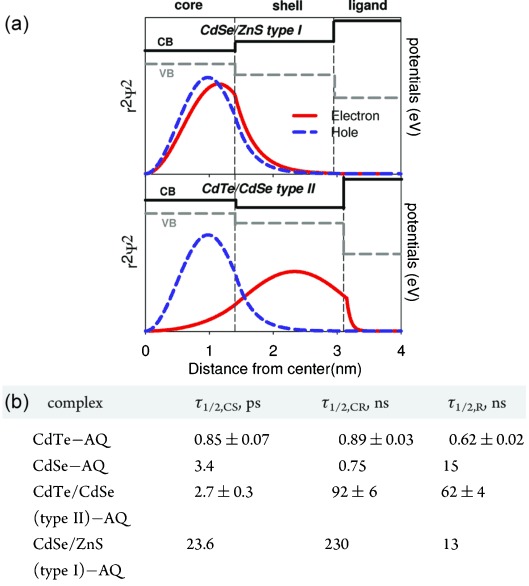
(a) CdSe/ZnS (type-I) and CdTe/CdSe (type-II) band-edge potentials and electron and hole radial probability density functions calculated for spherical core/shell nanocrystals. The type-I band alignment of CdSe/ZnS leads to the localization of both the electron and the hole in the CdSe core, whereas the type-II band alignment of CdTe/CdSe leads to the localization of the electron in the CdSe shell and the hole in the CdTe core. (b) Table of time constants for selected processes for the CdTe-AQ, CdSe-AQ, CdSe/ZnS-AQ, and CdTe/CdSe-AQ systems. The time constant for ET to AQ is denoted by *τ*_1/2,CS_, recombination of the electron in AQ with the hole in the nanocrystal is denoted by *τ*_1/2,CR_, and internal electron–hole recombination in the nanocrystals is denoted by *τ*_1/2,R_. Note that time constants are inverse of decay rates. The ratio of ET and internal electron–hole recombination rates, that is, *τ*_1/2,R_/*τ*_1/2,CS_, is largest for the type-II CdTe/CdSe nanocrystals. Adapted with permission from Ref. [86]. Copyright 2011, ACS.

Wavefunction engineering, achieved by epitaxial combination of two different semiconductors into one heterostructure nanocrystal,^68^,^69^,^128^,^129^ provides a method for controlling charge-transfer efficiencies. Nano-heterostructures can be designed to control internal electron–hole recombination rates and increase the probability density of a particular carrier at the nanocrystal surface. In core/shell heterostructures with type-I band alignment, where the valence and conduction band levels of the core material are sandwiched inside those of the shell, photoexcited electrons and holes are funneled to the core material (Figure 4a, top).^68^,^128^,^130^ The shell acts as a barrier to ET, as demonstrated for CdSe/CdS,^96^ ZnSe/ZnS,^95^ and CdSe/ZnS^85^,^97^ core/shell structures. In contrast, a type-II band alignment, where the valence and conduction band levels of the two semiconductors are staggered (Figure 4a, bottom), leads to relatively long-lived charge-separated states upon photoexcitation.^131^–^136^ This can increase the ratio of charge-transfer rate to the electron–hole recombination rate.^86^,^87^ This effect was demonstrated by a comparison of charge transfer and recombination dynamics between CdSe, CdTe, type-I CdSe/ZnS, and type-II CdTe/CdSe core/shell nanocrystals and the electron acceptor AQ (Figure 4).^86^ In Section 3, we describe how type-II nano-heterostructures are utilized to improve charge separation and increase the yields of photocatalytic reactions.

### 2.5. Feasibility of Nanocrystal Use in Photocatalysis

The experiments described in this section demonstrate that charge transfer out of nanocrystals, when compared with electron–hole recombination, can occur fast enough to make photocatalysis feasible. Electron–hole recombination rates in nanocrystals are typically in the range of 10^7^–10^9^ s^−1^.^27^,^90^,^93^ The charge-transfer rates from nanocrystals to acceptors, discussed above, cover a broad range, but are generally high.^137^ Rates of ET to adsorbed acceptors on the order of 10^9^–10^12^ s^−1^ have been reported.^82^,^85^,^86^,^97^,^98^,^101^,^109^ For covalently linked acceptors, those rates are 10^9^–10^10^ s^−1^.^106^,^109^ HT can take place on fast timescales as well, with reported rates ranging from 10^8^ to 10^11^ s^−1^.^102^–^104^,^116^ ET rates can even be competitive with exciton–exciton annihilation,^90^,^94^,^101^,^115^ which is usually observed with rates on the order of 10^10^–10^11^ s^−1^.^12^,^138^,^139^ Remarkably, hot-electron transfer from CdSe QDs to adsorbed MV^2+^ was also competitive with electron cooling,^97^ which occurs on a 10^12^ s^−1^ timescale.^140^–^142^ Such rapid ET rates suggest not only that charge transfer for photocatalytic reactions can be competitive with recombination processes, but also that, provided there is strong electronic coupling, even hot electrons and multiple excitons may be usable in photocatalysis.4

## 3. Photocatalytic Fuel Generation

A particularly intriguing use of nanocrystal photocatalysis is the harvesting of solar photons to drive chemical reactions that produce fuels. Solar fuel generation is an extremely challenging scientific and technological problem, with a high potential impact on our overall renewable energy portfolio.^143^–^146^ The highly tunable optical and surface properties of semiconductor nanocrystals make them potential candidates for the light-harvesting components of fuel-generating systems. The photoexcited carriers could then be delivered to redox co-catalysts, which are usually necessary to reduce the barriers for the multi-ET half-reactions involved in fuel generation.

In recent years, there have been several reports of colloidal II-VI nanocrystals used as components in photocatalytic systems for reactions such as H^+^ and CO_2_ reduction.^26^,^34^–^43^ These reactions follow a general scheme that involves (i) absorption of visible photons to produce photoexcited electrons and holes, (ii) transfer of electrons to reduction co-catalysts where they are utilized, and (iii) scavenging of holes to replenish the electrons used for reduction and to prevent nanocrystal degradation caused by the oxidizing holes. An energy level diagram for an example of this process is shown in Figure 7b below. As discussed in Section 2, the charge-transfer processes are in direct competition with electron–hole recombination. Nanocrystal–catalyst interactions that facilitate efficient ET are critical for the kinetics of the overall photochemical process. The ability of the catalyst to utilize the electrons delivered also plays a determining role. In this section, we review recent examples of fuel generation by nanocrystal photocatalysis. Our discussion is organized according to the nature of the co-catalyst employed, starting with Pt nanoparticles, continuing onto other inorganic co-catalysts, and ending with biological and bioinspired catalysts. As a means of comparison between the different systems described, we emphasize the quantum yield (QY) of the photocatalytic product. This quantity describes the ability of a given system to utilize absorbed photons for photocatalysis. It illustrates the competitiveness of excited-state processes that lead to fuel generation with electron–hole recombination pathways. The QY is not directly related to solar-power conversion efficiency because it does not take into account the fraction of the solar spectrum that can be absorbed and the amount of energy stored in the products.

**Figure 5:**
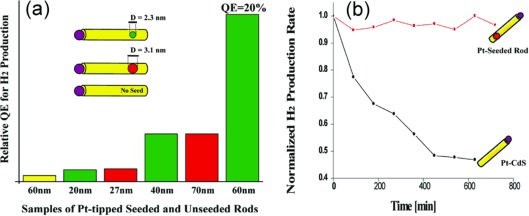
(a) A bar graph showing the relative quantum efficiencies of H_2_ production measured from nanorod-Pt photocatalytic systems. Unseeded CdS rods are shown in yellow, CdS rods with 3.1 nm CdSe seeds in red, and CdS rods with 2.3 nm CdSe seeds in green. The average CdS nanorod length is given on the *x* axis. The highest H_2_ production efficiency was achieved with the smallest seeds and longest seed–Pt distance due to the best charge separation in such structures. (b) Normalized H_2_ production over time from Pt-tipped seeded rods (red) and unseeded rods (black), illustrating improved stability when the CdSe seed was present. Reproduced with permission from Ref. [35]. Copyright 2010, ACS.

### 3.1. Nano-heterostructures Based on the CdS–Pt Interface

Photocatalytic H^+^ reduction using bulk CdS as the light absorber and Pt as the electron acceptor and reduction catalyst was first studied in the early 1980s.^60^,^147^,^148^ Analogous colloidal CdS–Pt nano-heterostructures, forming a direct, strongly coupled charge-transfer interface, can be synthesized by thermal or photochemical methods,^23^,^25^,^28^ as discussed in detail in Section 4. ET from photoexcited CdS to Pt is very efficient, as suggested by quenching of CdS PL^25^ and demonstrated by two recent direct measurements of charge-transfer dynamics.^26^,^27^ In Pt-decorated CdS nanorods obtained by photodeposition, ET occurred at rates faster than electron–hole recombination.^26^ More recently, charge-transfer dynamics were measured for CdS–Pt nano-heterostructures produced by thermal deposition of Pt on the ends of CdS nanorods.^27^ ET from photoexcited CdS to Pt was very fast (≈3 ps) and extremely competitive with electron–hole recombination. Furthermore, the charge-separated state was very long-lived (≈1 μs) because of hole trapping at the CdS surface, which reduced the electron–hole wavefunction overlap and therefore the recombination rate. Efficient and long-lived charge separation indicates that CdS–Pt structures are well suited for photocatalysis.

Amirav and Alivisatos demonstrated photocatalytic H_2_ generation with colloidal nanocrystals based on the CdS–Pt interface, using isopropanol as the hole scavenger (Figure 5).^35^ They compared the behavior of CdS nanorods and CdSe/CdS core/shell nanorods, both functionalized with one Pt nanoparticle. CdSe/CdS nanorods have type-II or quasi-type-II band alignment, depending on the size of the CdSe core.^149^–^153^ This results in localization of the photoexcited hole in the core and provides spatial separation between the hole and the electron, which quickly transfers to Pt. Thus, the charge-separated state in CdSe/CdS–Pt is expected to be longer-lived than that in CdS–Pt. As a consequence, CdSe/CdS–Pt nanocrystals produced significantly more H_2_ than CdS–Pt structures (Figure 5a). The highest QY of H_2_ production, 20 % (*λ*=450 nm), was recorded for CdSe/CdS–Pt structures that utilized a small CdSe core and had the furthest separation between CdSe and Pt components. The more quantum-confined CdSe has higher-lying conduction band levels, ensuring minimal electron density in the core. This effect, along with the spatial separation of the CdSe core from the Pt particle, is expected to provide particularly long-lived charge-separated states. Furthermore, core/shell-based heterostructures exhibited superior long-term stability during H_2_ generation (Figure 5b).^35^ This suggests that, although the photoexcited holes were localized in the CdSe core upon photoexcitation, they were scavenged by isopropanol between excitation events, preventing semiconductor degradation. A similar advantage of the CdSe/CdS core/shell nanorods was demonstrated in the example where ET from CdS to Pt was mediated by MV^2+^ to produce H_2_.^43^

In addition to nanocrystal properties, the size of the Pt nanoparticles plays an important role in photocatalysis.^23^ This was demonstrated for H_2_ generation mediated by CdS nanorods containing small Pt clusters deposited along the sidewall. Additional growth of a single 5 nm Pt particle did not improve the H_2_ yield, indicating that smaller particles which use less Pt may be better suited for photocatalysis. Similar utility of small Pt dots was observed for photocatalytic reduction of MB^+^ in CdSe–Pt structures.^89^

CdS nanorods decorated with Pt clusters were also used to ascertain the importance of hole-scavenging efficiency in solution.^33^ Four sacrificial electron donors were tested, listed here in order of increasing driving force for hole scavenging: methanol, disodium ethylenediaminetetraacetic acid, triethanolamine, and sodium sulfite. Both H_2_ generation efficiency and nanocrystal stability improved with increasing driving force.^33^ These results suggest that hole scavenging could be engineered and controlled to maximize the photocatalytic capacity of a given nanocrystal–catalyst system.

**Figure 6:**
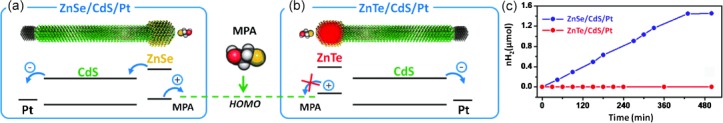
Energy level diagrams and schematic representations of charge-transfer processes in semiconductor–metal nano-heterostructures containing (a) ZnSe/CdS/Pt and (b) ZnTe/CdS/Pt. A comparison of (a) and (b) illustrates that a hole in ZnTe is less energetic than the one in ZnSe, and is unable to oxidize the surface ligand MPA. (c) H_2_ production using ZnSe/CdS/Pt (blue) and ZnTe/CdS/Pt (red) nano-heterostructures, showing that heterostructures which cannot oxidize the surface ligand produce significantly less H_2_. Reproduced with permission from Ref. [36]. Copyright 2011, ACS.

Hole scavenging can even be carried out by the nanocrystal surface-capping ligands. This was demonstrated in the example of ZnX/CdS nanocrystals, where X=Se or Te, functionalized with a Pt tip at the CdS end and capped with mercaptocarboxylic acid ligands (Figure 6).^36^ ZnSe/CdS and ZnTe/CdS are type-II heterostructures, favoring photoexcited electron density on CdS (and subsequently Pt), and hole localization in ZnX. Photocatalytic H_2_ generation was carried out in an 8 : 1 water/methanol mixture, so that the alcohol could serve as a hole scavenger. While ZnSe/CdS/Pt structures were capable of significant H_2_ generation, ZnTe/CdS/Pt nanocrystals with the same surface-capping ligands produced several orders of magnitude less H_2_ (Figure 6c). This difference was attributed to the role of the surface-capping ligand as a more efficient hole scavenger than methanol. The holes in the ZnTe cores were not energetic enough to transfer to the ligand (Figure 6b). In accordance with this hypothesis, after the photocatalysis rate slowed for the ZnSe/CdS/Pt nanocrystals, an addition of excess ligand molecules to the nanocrystal solution resulted in recovered H_2_ generation rates.

### 3.2. Nanocrystals with Alternative Inorganic Catalysts

With the high cost of Pt as a motivation to reduce its use, efforts are emerging to integrate nanocrystals with alternative inorganic catalysts. Pd-based materials are catalysts for methane combustion, alcohol oxidation, and water reduction.^154^–^156^ CdS nanorods functionalized with Pd_4_S and PdO have demonstrated photocatalytic H_2_ production.^40^ CdS–Pd_4_S structures were formed by a combination of metal reduction and cation exchange, resulting in Pd_4_S regions inside the nanorod. In contrast, PdO islands nucleated heterogeneously along the nanorod sidewalls. Both types of structures produced H_2_ with QY values in the single digits,^40^ comparable to those previously measured for CdS–Pt.^35^ Molybdenum sulfide species have also been explored as co-catalysts for CdSe/CdS type-II nanorods because of their H^+^ reduction catalytic activity and relatively low cost.^42^ Nanorods coated with amorphous MoS_3_ produced H_2_ with an apparent QY of 10 % (*λ*=450 nm), which was about half of the efficiency measured for Pt-tipped CdSe/CdS.^35^ These examples illustrate that increased efforts to identify inexpensive co-catalysts to serve as alternatives to Pt may lead to exciting new materials for photochemical fuel generation.

There are also several examples of photocatalytic systems based on CdSe, rather than CdS, which produce H_2_.^157^–^160^ For example, quantum-confined CdSe QDs with Cd metal clusters on their surfaces are capable of photocatalytic H_2_ generation with sodium sulfite as a hole scavenger.^159^ Remarkably, the dependence of H_2_ production on CdSe size, and therefore, the conduction-band energy and Δ*G* for H^+^ reduction, followed the exponential dependence expected from Marcus theory. This is consistent with the results described in Section 2.3. Another unique example is that of extremely quantum-confined CdSe nanoribbons with a band gap of 2.7 eV, which can produce H_2_ even without a co-catalyst, with a QY of 9 % (*λ*=440 nm). Use of an MoS_2_ co-catalyst notably increased the photocatalytic activity. Interestingly, no improvement over CdSe alone was observed with a Pt co-catalyst because Pt was poisoned by free sulfide and Se in solution.^158^ This observation suggests that sulfide-based co-catalysts may be better suited for a chalcogenide-rich environment where catalyst poisoning is a concern.

### 3.3. Biomimetic Nanocrystal–Catalyst Assemblies

Photosynthesis – nature’s process for storing solar energy inside chemical bonds – provides inspiration and some design principles for solar fuel generation.^161^–^164^ In photosynthesis, light absorption and catalysis are performed by light-harvesting proteins and enzymes, respectively, and the two processes are coupled through a finely tuned series of ET steps. While energy conversion efficiencies of natural photosynthesis are relatively low,^161^ coupling excellent light absorbers, such as nanocrystals, to fast enzymatic catalysts that can utilize photoexcited charges may teach us how to design more efficient artificial systems. Hydrogenases, formate dehydrogenases, and CO dehydrogenases catalyze fuel-forming reactions with high selectivities.^163^,^165^–^178^ Herein, we describe how nanocrystals and enzymes have been combined for photocatalytic fuel generation. In addition to photocatalytic QY, we discuss two more metrics that are commonly used in enzyme catalysis: turnover frequency (TOF) and turnover number (TON). The former denotes the rate of product formation and is indicative of catalytic efficiency upon delivery of electrons to the enzyme, whereas the latter quantifies how many turnovers the catalyst achieves before ceasing activity.

King and co-workers described a biomimetic approach for coupling [FeFe]-hydrogenase from *Clostridium acetobutylicum* (CaI) to CdTe nanocrystals.^37^ CaI is capable of operating at very high TOFs, up to 21000 H_2_ molecules per enzyme per second.^179^ In vivo, electron delivery to CaI occurs via the redox-shuttle ferredoxin, which docks in a positively charged pocket on the enzyme.^180^,^181^ A similar electrostatic interaction between CdTe and CaI was enabled by the use of the 3-mercaptopropionate (MPA) ligand, which attached to the nanocrystals through thiolate groups and presented negatively charged carboxylate groups to the enzyme. Upon illumination of these complexes with visible light, ET from CdTe to CaI led to H_2_ production, while the holes were scavenged by ascorbate in solution. The QY of H_2_ generation was 9 % (*λ*=532 nm) under optimized sample conditions.

**Figure 7:**
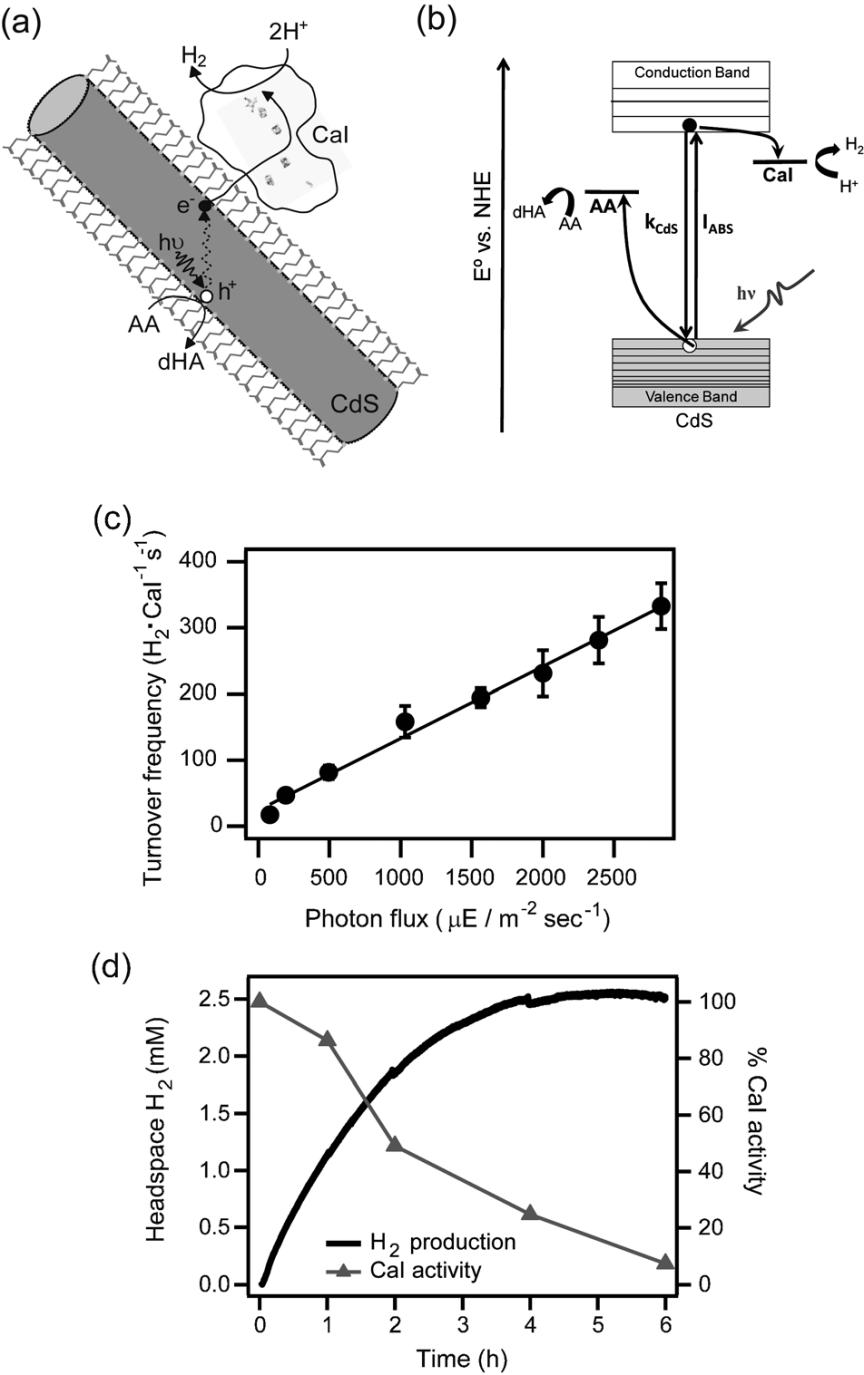
(a) Schematic representation of photocatalytic H_2_ production by CdS–CaI complexes. CdS and CaI are drawn to scale, while MPA molecules are enlarged by about five-fold. (b) Energy level diagram for H_2_ generation. Photoexcited electrons transfer to CaI, while the holes are scavenged by ascorbate (AA). The competing electron–hole recombination pathways are denoted as *k*_CdS_. (c) H_2_ generation rate, expressed as a TOF, has a linear dependence on 405 nm photon flux. (d) Total H_2_ generated over time (solid line) and the corresponding relative CaI activity values (triangles). The data illustrates enzyme deactivation during H_2_ production. Adapted with permission from Ref. [38]. Copyright 2012, ACS.

Further mechanistic insights into photochemical H_2_ production by nanocrystal–CaI complexes were derived from a study of CdS nanorod–CaI biohybrids (Figure 7).^38^ These heterostructures reduced H^+^ to H_2_ with a QY of 20 % (*λ*=405 nm). This value is an order of magnitude higher than the QY of H_2_ generation measured for similar CdS nanorods with Pt as the co-catalyst (Figure 5a).^35^ This increase may be due to the high efficiency and catalytic selectivity of CaI. H_2_ production efficiency by the CdS–CaI heterostructures was linear with photon flux (Figure 7c). This indicated that H_2_ generation was limited by the availability of electrons (photon flux×efficiency of ET), and not by the turnover capacity of the enzyme. H_2_ production rate slowed after 30 min and ceased after 4 h (Figure 7d), with the overall TON on the order of 10^6^. Cessation was attributed to deactivation of the enzyme by small amounts of free MPA molecules photo-oxidized off the nanocrystal surface (Figure 7d). CdS nanorods were not significantly changed during H_2_ generation and neither precipitation nor degradation of nanocrystals was observed.

[NiFe]-hydrogenases are another catalytically interesting family of enzymes because, while naturally less efficient for H^+^ reduction, they are more O_2_ tolerant than the [FeFe] family.^167^,^172^–^175^,^182^,^183^ Complexes of [NiFe]-hydrogenase from *Thiocapsa roseopersicina* (Tr) electrostatically coupled to MPA-capped CdTe QDs produced H_2_ under visible light.^41^ The maximum QY of H_2_ production was 4 % (*λ*=527 nm) with a TON of 92. Some of the reduced efficiency and lower TON, when compared with nanocrystal–CaI systems, can be attributed to the catalytic bias of [NiFe]-hydrogenases in the direction of H_2_ oxidation. Insights into the utilization of photoexcited electrons by the enzyme were obtained from an FTIR study of the catalytic states under H_2_ production conditions. Accumulation of redox intermediates associated with slow turnover was not observed, indicating that ET from CdTe to the enzyme active site was conducive to efficient catalysis. This suggested that the overall yield of H_2_ was limited by the availability of electrons and the competitiveness of the ET process with recombination pathways.

In addition to natural catalysts, molecular mimics of the hydrogenase active site are being investigated as H^+^ reduction catalysts that could be synthetically mass-produced.^184^–^188^ Molecules containing an artificial [FeFe] active site have been coupled with CdTe nanocrystals for photocatalytic H_2_ generation.^39^ The optimized system produced H_2_ with a TOF of 0.83 s^−1^ and a TON of 505. Although these numbers are significantly lower than those measured for the natural [FeFe]-hydrogenase,^38^ they are roughly consistent with the much lower TOF capabilities of the molecular mimics.^184^–^188^

In addition to H_2_ generation, nanocrystal–enzyme complexes have been used to photocatalyze the more catalytically challenging CO_2_ reduction reaction.^34^,^164^ Carbon monoxide dehydrogenases (CODHs) catalyze the interconversion of CO_2_ and CO at high TOF, while bypassing a challenging intermediate product.^34^,^168^ Complexes of *Carboxydothermus hydrogenoformans* CODH with CdS nanocrystals reduced CO_2_ to CO under visible illumination.^34^ The average photoreduction TOF for CODH coupled to CdS nanorods was about 1 s^−1^, which is a relatively high value for a photocatalytic CO_2_ reduction, but well below the maximum capability of the enzyme. TON values were about 10^4^ for assemblies of CODH with both nanorods and QDs. The photocatalytic activity of this system appeared limited by the ability to deliver electrons from photoexcited CdS because of the competing recombination of photogenerated charges.

A general trend has emerged for nanocrystal–enzyme complexes, suggesting that photocatalytic efficiency is usually not limited by the turnover capacity of the enzyme, but rather by electron flux from the nanocrystal to the enzyme active site. This suggests that improvements can be achieved by maximizing ET efficiencies through increased electronic coupling between the two components and the use of type-II nanocrystals with long-lived excited states.

## 4. Photochemical Deposition of Metals on Colloidal Semiconductor Nanocrystals

Photocatalytic synthetic reactions, which reduce and oxidize species in solution to deposit new material onto a nanocrystal, may lead to unique products and provide a new synthetic methodology. In the last five years, there have been several reports of photochemical deposition of nanoparticles of metals, such as Pt, Pd, and Au, on CdS and CdSe/CdS nanocrystals.^21^–^25^ The basic photochemical reaction scheme follows the three-reactant approach used on CdS powders in the 1980s: light-absorbing semiconductor; molecular precursor containing the metal in the oxidized form; and an electron donor, such as an alcohol or an amine.^147^,^189^,^190^ Photoexcited electrons reduce the metal, depositing it on the semiconductor surface, while the holes oxidize the sacrificial electron donor. The mechanisms of such reactions are likely to be more kinetically complicated than those of the model reactions described in Section 2 because they involve multiple charge-transfer events. Herein we briefly describe recent examples of photochemical metal deposition on CdS and CdSe/CdS nanocrystals, with special emphasis on mechanistic insights such as factors that determine the location of the metal deposits and the contrast between photochemical and thermal reactions.

### 4.1. Photodeposition of Pt and Pd on CdS- and CdSe-Based Nanocrystals

**Figure 8:**
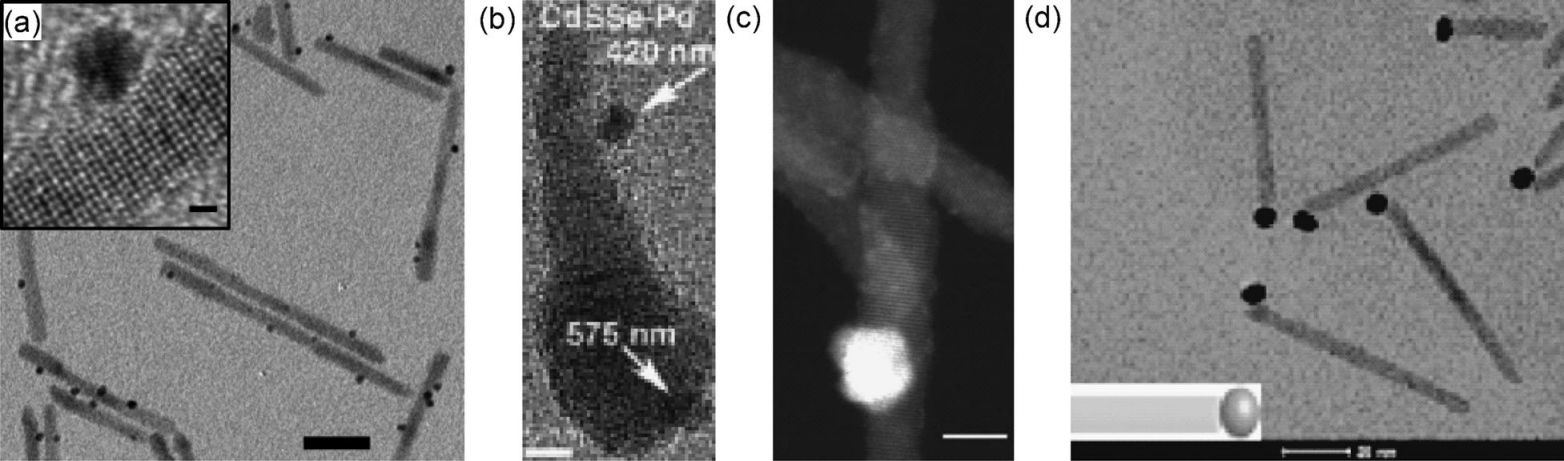
(a) TEM image of CdS–Pt nano-heterostructures synthesized by photochemical deposition of Pt on CdS nanorods. Scale bar: 20 nm. Inset: high-resolution TEM image of a nanorod section and the attached particle. Scale bar: 1 nm. Adapted with permission from Ref. [25]. Copyright 2008, Wiley. (b) Results of photodeposition of Pd on teardrop-shaped CdSe/CdS nanostructures. With higher excitation wavelengths, nucleation near the thick CdSe-rich regions is more common, while lower excitation wavelengths led to preferential nucleation near the thin CdS-rich regions. Scale bar: 5 nm. Reproduced with permission from Ref. [22]. Copyright 2011, ACS. (c) HAADF-TEM image of structures resulting from photodeposition of Pt on water-soluble CdS nanorods. The image shows one Pt nanoparticle per nanorod and many small Pt clusters. Scale bar: 5 nm. Reproduced with permission from Ref. [29]. Copyright 2011, Wiley. (d) TEM image of CdS–Au nano-heterostructures resulting from photochemical deposition of Au on CdS nanorods with suppression of thermal reactions. Scale bar: 20 nm. Reproduced with permission from Ref. [21]. Copyright 2009, ACS.

In 2008, Alivisatos and co-workers reported the photochemical deposition of Pt nanoparticles on colloidal CdS and CdSe/CdS core/shell nanorods capped with long-chain phosphonate surface-coordinating ligands.^25^ The reaction utilized an organic-soluble Pt precursor (1,5-cyclooctadiene)dimethylplatinum(II), while tertiary amines were used as hole scavengers. The illumination wavelength was chosen to excite only CdS and prevent homogeneous nucleation of Pt particles. Exposure to 458 nm light resulted in deposition of small Pt nanoparticles (diameter <3 nm) distributed heterogeneously along the nanorod length (Figure 8a). Unlike the one-electron charge-transfer processes described in Section 2, reduction of Pt^II^ to Pt^0^ with concurrent amine oxidation is kinetically complicated, and therefore, relatively slow. For that reason, nucleation of Pt particles occurred at the locations of relatively long-lived carrier trap states on the nanocrystal surface (e.g., unpassivated Cd^2+^ sites). Subsequent growth of particles occurred by fast ET from CdS to the Pt islands, followed by reduction of additional precursor. This mechanism illustrates the importance of the locations (i.e., probability densities) of the photoexcited electrons and holes in determining the final product.

The photochemical metal deposition mechanism is in stark contrast to thermal deposition of Pt on CdS nanorods.^28^ In the thermal reaction, the metal precursor is decomposed in the presence of CdS nanorods and a reducing agent at 200 °C, resulting in Pt particle growth at the nanorod ends. Such growth is attributed to preferential metal nucleation on the end facets, which have higher surface energies due to less complete passivation.^71^

A similar photochemical approach has been used for deposition of Pt and Pd nanoparticles on both CdS nanorods and teardrop-shaped CdS_1-*x*_Se_*x*_ nanocrystals (Figure 8b).^22^ The latter structures had a graded composition ranging from CdSe-rich at the thick end to CdS-rich at the thin end.^30^ Pd photodeposition on these nanocrystals showed notable excitation wavelength dependence. When illuminated at wavelengths that could be absorbed by both CdS and CdSe, Pt deposition was selective for the CdS-rich ends of the nanocrystals. This was attributed to a higher concentration of surface trap states at the thinner CdS ends. With longer wavelengths absorbed mostly by CdSe, there was a strong preference for metal deposition near CdSe-rich regions. This example supports the notion that photochemical metal deposition reactions are governed by the probability distributions of the photoexcited electrons and holes.

A somewhat different mechanistic pathway was observed in the case of Pt photodeposition on CdS nanorods under aqueous conditions (Figure 8c).^23^ Water-soluble CdS nanorods were obtained by ligand exchange from phosphonic-acid capped nanostructures to d-,l-cysteine hydrochloride. The metal precursor was H_2_PtCl_6_, while both triethanolamine and ascorbic acid were used as electron donors. In the initial stages of photodeposition, small Pt clusters formed along the nanorod length. At later stages, in the presence of ascorbic acid, an additional single Pt nanoparticle was observed per nanorod. The formation of the single Pt nanoparticle was attributed to one metal cluster on each nanorod randomly attracting more photoexcited CdS electrons than the other clusters. This resulted in further Pt deposition onto that cluster, with preferential ET amplified as the cluster grew into a particle. The use of these particles for photocatalytic H_2_ generation was described in Section 3.

### 4.2. Photochemical and Thermal Au Deposition Pathways on CdS and CdSe/CdS Nanocrystals

Banin and co-workers described an explicit comparison of thermal and photochemical pathways in the deposition of Au on CdS and CdSe/CdS core/shell nanorods.^21^ Phosphonic-acid capped nanocrystals were mixed with AuCl_3_, organic amines, and dodecyldimethylammonium bromide in toluene. The amines serve both as surface-capping ligands and as hole scavengers. Thermal reduction of Au^3+^ resulted in small Au nanoparticles along the nanorod length, which nucleated as a result of defects induced by missing surface-capping ligand molecules.^31^ This thermal growth could be suppressed by lowering the reaction temperature, causing the ligands to be less dynamic, their surface coverage to increase, and access of Au precursor to the nanocrystal surface to be inhibited. Illumination resulted in additional formation of one large Au nanoparticle at the sulfur-rich end of each nanorod. This site specificity is attributed to the attraction of the positively charged Au precursor to the negative sulfur dangling bonds, and to the strong driving force for the formation of an Au–S bond. Additionally, photoexcited CdS electrons may preferentially transfer to the specific Au particle located at the nanorod end.^24^ With an Au particle present at the nanorod end, further growth occurred through ET from the photoexcited nanocrystal to the Au island. Reducing the temperature of the photochemical reaction to suppress the thermal pathway resulted in particles with only one Au nanoparticle at the nanorod end (Figure 8d).^21^

It is noteworthy that, in the case of Au, the thermal pathways deposit the metal on the nanorod sides and photochemical reaction locates the metal at the nanorod end, while the opposite is true for Pt deposition. The reason for this contrast is unclear, but it may be related to the particular strength of the Au–S bond and the existence of a sulfur-rich facet on one of the nanorod ends. Nevertheless, the examples of photochemical metal deposition described above demonstrate the potential of nanocrystal photocatalysis to serve as a synthetic tool complementary to thermal methods.

## 5. Summary and Outlook

In this review, we described recent progress in the photocatalysis of II-VI semiconductor nanocrystals and some of the guiding principles emerging from the expanding literature on the subject. Measurements of charge-transfer rates from photoexcited nanocrystals to directly adsorbed or chemically linked electron/hole acceptors demonstrate that extraction of the photoexcited charges can be competitive with recombination pathways. The rates and efficiencies of charge transfer can be controlled through donor–acceptor electronic coupling and the driving force for the process. Both parameters are synthetically tunable. Type-II nano-heterostructures with long-lived charge-separated states can improve the kinetic competitiveness of charge-transfer processes, as seen in both the case of charge transfer to redox dyes and photocatalytic H_2_ generation.

Nanocrystals can mediate H_2_ production under visible irradiation when coupled with inorganic or biological and bioinspired co-catalysts. Reasonably high QYs of H_2_ generation indicate that the processes that lead to H^+^ reduction can be competitive with electron–hole recombination and other decay pathways. Long-lived charge-separated states in type-II nano-heterostructures can improve both H_2_ generation QY and long-term photocatalytic activity. In nanocrystal–enzyme complexes, the largest gains may be achieved by improving electronic coupling to increase the efficiency of ET between the light absorber and catalyst.

Considering the observations made to date about fuel generation mediated by nanocrystals, we know little about the kinetic bottlenecks that make photocatalytic processes less efficient than the very fast charge transfer observed for one-electron processes. The overall photocatalytic rate is a delicate balance of the rates of excitation, charge transfer, recombination, electron utilization by the catalyst, back-ET, and hole scavenging. Time-resolved spectroscopy may provide a means of deconvoluting these various rates. Perhaps most pressingly, the behavior of photoexcited holes is far from understood. HT is critical in three ways: as the process that replaces the electrons transferred from the nanocrystal, as the means of preventing semiconductor degradation, and as a way to perform the oxidation half-reaction. It would be desirable to learn how to couple nanocrystals to oxidation catalysts such that photoexcited holes could be quickly removed from the semiconductor and used for processes such as water oxidation. Two different nanocrystalline materials could be coupled into a Z-scheme analogous to photosynthesis. In addition, information is needed about back-reactions of intermediates or recombination of products on these very small particles. Challenges notwithstanding, nanocrystals are remarkably tunable through synthesis, are well-defined soluble crystalline materials, and are amenable to spectroscopic studies of processes involved in photocatalysis. Thus, they present an excellent model system to understand the kinetic issues involved in complicated light-driven reactions.

Finally, we described deposition of metals on semiconductor nanocrystals as another application of nanocrystal photocatalysis. Because these photochemical reactions are guided by the probability distributions of photoexcited carriers and the locations at which they are trapped, their products can be quite different from those of thermal deposition reactions. While our current understanding of how to control photochemical synthetic reactions is rudimentary, further efforts in this area could lead to the ability to create more complex nano-heterostructures. Ideally, the reach of this synthetic methodology will go beyond metal reduction and include oxidation processes or more complex reactions to deposit semiconductors or oxides.

## Figures and Tables

**Figure:**
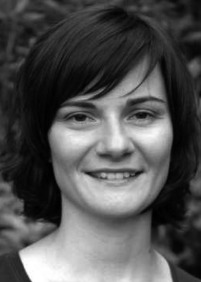


**Figure:**
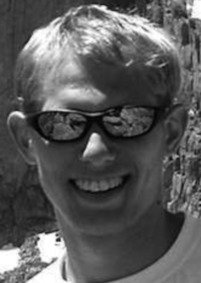


**Figure:**
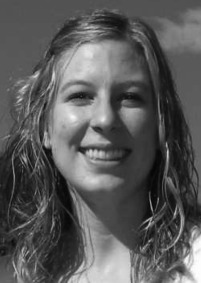


## Biographical Information

Molly B. Wilker received her undergraduate degree in chemistry from Gustavus Adolphus College in St. Peter, MN. She is currently a PhD student in physical chemistry at the University of Colorado Boulder. Her research focuses on the integration of nanocrystals with redox catalysts.

Kyle J. Schnitzenbaumer earned a Bachelor of Science degree in chemistry from Illinois Wesleyan University in Bloomington, IL. He is currently a physical chemistry PhD student at the University of Colorado Boulder. His research interests are centered on the photophysics and photochemistry of semiconductor nanocrystals.

Gordana Dukovic received her PhD in physical chemistry from Columbia University with Louis E. Brus. She was subsequently a postdoctoral scholar at the University of California Berkeley with A. Paul Alivisatos. She is currently an Assistant Professor in the Department of Chemistry and Biochemistry at the University of Colorado Boulder. Her research interests are in the synthesis and spectroscopy of nanoscale materials, with a focus on processes and materials relevant to solar-energy harvesting.
